# Editorial: Vitamin D and mineral ion homeostasis: endocrine dysregulation in chronic diseases

**DOI:** 10.3389/fendo.2025.1493986

**Published:** 2025-01-21

**Authors:** Rahnuma Ahmad, Bara Sarraj, Mohammed S. Razzaque

**Affiliations:** ^1^ Department of Physiology, Medical College for Women and Hospital, Dhaka, Bangladesh; ^2^ Department of Biological Sciences, Harold Washington College Chicago, Chicago, IL, United States; ^3^ Department of Medical Education, School of Medicine, University of Texas Rio Grande Valley (UTRGV), Edinburg, TX, United States

**Keywords:** vitamin D, parathyroid hormone, calcium, magnesium, potassium

This ‘Research Topic’ is intended to bring together experts to share their experiences in explaining the roles and regulations of vitamin D and mineral ions in various chronic human diseases. A total of 15 articles by 102 authors have been published in this ‘Research Topic’ to accomplish the intended objectives. Seven of those published articles detail various functional aspects of vitamin D, two articles explain kidney stone-related complications, another two articles discuss parathyroid pathology, and the remaining articles elaborate mineral ion dysregulation in various disease pathologies. Micronutrients, including mineral ions and trace elements, work together to optimize the biological and biochemical functions of the body. Essential components such as calcium, phosphate, zinc, iron, selenium and magnesium, as well as vitamins, play crucial roles in maintaining metabolic balance within the body. Delicate interactions of these nutrients are vital for the physiological functioning of various systems and organs.

Liu et al. studied the relationships of serum 25(OH)D levels with blood pressure and glucose metabolism. They reported that low 25(OH)D levels are correlated with increased diastolic blood pressure, HbA1c, and triglycerides and decreased HDL-C. Vitamin D deficiency may impair glucose tolerance by reducing calcium levels, insulin secretion, and beta-cell function. Vitamin D also suppresses renin synthesis to lower blood pressure. Vitamin D deficiency increases PTH, leading to arterial stiffness and increased lipid factor levels through the expression of receptor for advanced glycation end products (RAGE) and cytokine production (1). Li et al. summarized the vitamin D-diabetes relationship. Studies have shown that serum 25(OH)D levels are negatively correlated with type 2 diabetes risk. Vitamin D supplementation reduces diabetes risk, especially in prediabetic individuals, improves glucose tolerance, and decreases the risk of complications. However, the benefits may be limited to nonobese patients (2). Vitamin D deficiency has been associated with endothelial dysfunction and atherosclerosis. These vascular changes may result in nephropathy and alterations in renal function, particularly in diabetic patients (3).

Hu and Yang studied U.S. vitamin D trends via NHANES data from 2001–2018 and reported that serum 25(OH)D levels increased among adults, with an L-shaped correlation between vitamin D levels and cardiovascular disease (CVD)/all-cause mortality. Levels below 50 nmol/L were associated with increased CVD and mortality risk. Improved awareness, living standards, dietary inclusion, and supplementation likely contributed to better vitamin D status. The study emphasized the need for continued awareness to prevent vitamin D deficiency. Vitamin D plays an immunomodulatory role, stimulating innate immunity and suppressing acquired immunity. 25(OH)D levels are correlated with autoimmune antibody titers, including those related to thyroid function (4).

Vitamin D has a U-shaped effect on both inflammation and calcium-phosphate metabolism. This was recently demonstrated for 25(OH)D and 1,25(OH)_2_D in a large cross-sectional study (5). Consequently, it is not surprising that vitamin D supplementation does not benefit everyone. Clear benefits from vitamin D supplementation are observed in patients with vitamin D deficiency (6, 7). However, patients who already have adequate vitamin D levels usually do not benefit from additional supplementation. This highlights the need for the status of vitamin D assessment in chronic kidney disease (CKD) patients to avoid both under- and over-dosing of supplementary vitamin D. The recent study by Li et al. provides useful targets for both 25(OH)D and 1,25(OH)_2_D levels (5).

Yu et al. investigated the causal link between vitamin D supplementation and autoimmune thyroid disease (AITD) prognosis. They reported that a) higher serum vitamin D levels correlate with reduced AITD risk and that b) vitamin D may inhibit AITD by suppressing T-cell activation, increasing Tregs, inhibiting naïve T-cell differentiation, and reducing HLA II gene expression. While vitamin D is essential for many physiological functions, excess vitamin D can cause severe hypercalcemia, leading to symptoms such as confusion, vomiting, abdominal pain, and polyuria. Although rare, vitamin D toxicity can result from long-term overconsumption, metabolic pathway malfunction, or diseases that cause overproduction of active vitamin D metabolites (8). Xing et al. compared venous and capillary blood collection methods for 25(OH)D detection via a chemiluminescence immunoassay (CLIA): a) venous blood yielded higher 25(OH)D values than capillary blood did, b) capillary blood testing is useful when venous collection is challenging (e.g., obesity, burns, cancer, children), and c) they recommend the use of a truncation value from a linear equation for vitamin D status assessment. Despite global vitamin D deficiency concerns, testing is expensive and not recommended for the general population. The Endocrine Society suggests screening only at-risk individuals. Consequently, a tool for identifying those at risk of vitamin D deficiency is needed to optimize screening efforts. Guo et al. developed a cost-effective tool to predict vitamin D deficiency (<50 nmol/L) via machine learning: a) used easily collectable community data, b) employed the XGBoost method in an online web calculator, and c) allowed clinicians to avoid unnecessary vitamin D testing.

PTH is essential for calcium homeostasis and vitamin D activation. However, thyroidectomy patients are at risk of accidental parathyroid gland removal because of their location behind the thyroid poles (9). Leszczyńska et al. reported a rare case in which a 58-year-old female who underwent parathyroidectomy developed recurrent hypercalcemia 2 years after vitamin D supplementation. Tests revealed suppressed PTH, high serum calcium, and elevated 1,25(OH)_2_D. A low 24,25(OH)D and high 25(OH)D/24,25(OH)D ratio indicate vitamin D catabolism defects. The patient had a CYP24A1 gene mutation, affecting the 24-hydroxylase enzyme for vitamin D catabolism. Díez et al. compared the development of various comorbidities in patients suffering from long-term hypoparathyroidism resulting from thyroidectomy with comorbidities in subjects without hypoparathyroidism following thyroidectomy. Those authors noted that those with hypoparathyroidism have a greater risk of suffering from chronic kidney disease, cardiovascular disease, and nephrolithiasis. However, these patients have a lower risk of incident fractures. Disease of the kidney may be due to hypercalciuria with the formation of calcium phosphate deposits and their deposition in the renal tubules. Hypocalcemia and PTH deficiency at the vascular and cardiac levels may lead to cardiovascular complications (10). On the other hand, hypercalcemia with hypocalciuria may also result in symptoms such as fatigue, weakness, increased risk of coronary heart disease, chronic kidney disease, chondrocalcinosis, pancreatitis, and femoral fractures (11).

The circadian rhythm affects vitamin D and PTH homeostasis: vitamin D shows diurnal variation, decreases in the morning and plateaus during the day (12). Vitamin D deficiency is linked to inadequate sleep or abnormal light exposure. Night shift workers have lower serum 25(OH)D levels than day workers do because of reduced sunlight exposure (13). He et al. conducted a review in which they highlighted those conditions such as hypertension, metabolic syndrome, microbiome dysbiosis, inflammatory bowel syndrome, vitamin D deficiency, and PTH disorders were related to a disruption in the circadian clock. Each of these diseases also causes the development of kidney stone disease. Oxidative stress, insulin resistance, calcium metabolism disorders, high blood lipid levels, and inflammation may be the underlying pathologies of kidney stone development due to these diseases.

Liu et al. summarized the potential role of magnesium in osteoporosis. Magnesium inadequacy can disrupt the regulation of parathyroid hormone (PTH) and vitamin D, which in turn affects the RANK/RANKL/OPG signaling pathway. This dysregulation results in increased osteoclastic activity, contributing to bone loss and the development of osteoporosis (14). Studies indicate that magnesium supplementation can increase bone density and prevent further bone loss; neuroprotective effects of magnesium in cognitive decline is also reported (15). Therefore, magnesium supplementation represents an easy and cost-effective strategy to delay the progression of osteoporosis, particularly in elderly individuals (16).

Li et al. investigated the potential association between nonalcoholic fatty liver disease (NAFLD) and kidney stone formation. Although they reported no significant link between the two, they proposed that mechanisms such as oxidative stress, insulin resistance, lipotoxicity, and inflammation could contribute to kidney stone formation in individuals with NAFLD. Additionally, elevated blood lipid levels may lead to hyperuricemia. Liu et al. reported that adiposity markers are correlated with hyperuricemia. Lipid parameters strongly predict hyperuricemia, especially in women. Elevated triglycerides and lipid metabolism disorders may impair renal function, reducing uric acid excretion and causing hyperuricemia. Knowing the levels of serum vitamin D is crucial for individuals with metabolic, cardiovascular, autoimmune, and bone disorders. Both venous and capillary blood can be used for testing (17). Zhang et al. reported a correlation between renal function and vascular damage in the carotid artery among individuals with type 2 diabetes mellitus. Specifically, serum creatinine levels were positively correlated with carotid artery damage, whereas the estimated glomerular filtration rate (eGFR) was negatively correlated with carotid artery atherosclerosis.

Familial hypocalciuric hypercalcemia (FHH) is characterized by increased serum calcium, a normal to high concentration of PTH, and hypocalciuria and is an autosomal disorder. This occurs due to genetic mutation. Lin et al. investigated the genetic cause of FHH. They reported that FHH is caused by a mutation in the CASR gene; the mutation is a *de novo* heterozygous mutation. The specific mutation is c. T1661A,1554 N; this mutation occurs in the cysteine-rich domain of the CASR gene. This finding helps characterize the genetic basis of FHH, providing insight into its development and potential diagnostic markers.

Sun et al. conducted a study examining the relationships among dietary potassium intake, serum potassium levels, and survival in hemodialysis patients, both with and without dietary potassium restrictions. They reported that plant-based foods high in potassium, such as potatoes and melons, also contain significant carbohydrates that can lower plasma potassium levels through insulin release. Animal-based foods are high in potassium but low in carbohydrates, leading to elevated serum potassium levels. In hemodialysis patients, potassium excretion occurs primarily through feces (18). Meat consumption can worsen uremia and cause constipation through the formation of nitride-containing products, whereas a plant-based diet may help reduce uremic toxins through increased fiber intake (19). The authors concluded that there is little to no direct association between dietary potassium and serum potassium levels in these patients. They recommended that dietary considerations should focus not only on potassium content but also on the type of food and its overall nutrient profile.

The articles published in this Research Topic highlighted the significant clinical and biological roles of various minerals and vitamins in maintaining metabolic balance. Minerals and vitamins play crucial roles in maintaining overall health, and proper metabolic balance depends on adequate levels of these nutrients. Additional research into diseases related to mineral ion metabolism is needed to gain a deeper understanding of the conditions associated with vitamin D and mineral ion dysregulation (20–24). Additionally, identifying populations at risk for nutrient deficiencies and encouraging the consumption of diets rich in minerals and vitamins to potentially delay the onset of associated diseases will open new avenues for preventive medicine. Finally, this Research Topic provides valuable insights while also highlighting areas where more research is needed to fully understand the complex relationships between nutrients and health.
